# A novel role for lncRNAs in cell cycle control during stress adaptation

**DOI:** 10.1007/s00294-014-0453-y

**Published:** 2014-09-28

**Authors:** Carme Solé, Mariona Nadal-Ribelles, Eulàlia de Nadal, Francesc Posas

**Affiliations:** Cell Signaling unit, Departament de Ciències Experimentals i de la Salut, Cell Signaling Research Group, Universitat Pompeu Fabra (UPF), Dr Aiguader 88, E-08003 Barcelona, Spain

**Keywords:** SAPKs, Hog1, Osmostress, LncRNA, Gene expression, Cell cycle

## Abstract

Eukaryotic cells have developed sophisticated systems to constantly monitor changes in the extracellular environment and to orchestrate a proper cellular response. To maximize survival, cells delay cell-cycle progression in response to environmental changes. In response to extracellular insults, stress-activated protein kinases (SAPKs) modulate cell-cycle progression and gene expression. In yeast, osmostress induces activation of the p38-related SAPK Hog1, which plays a key role in reprogramming gene expression upon osmostress. Genomic analysis has revealed the existence of a large number of long non-coding RNAs (lncRNAs) with different functions in a variety of organisms, including yeast. Upon osmostress, hundreds of lncRNAs are induced by the SAPK p38/Hog1. One gene that expresses Hog1-dependent lncRNA in an antisense orientation is the *CDC28* gene, which encodes CDK1 kinase that controls the cell cycle in yeast. Cdc28 lncRNA mediates the induction of *CDC28* expression and this increase in the level of Cdc28 results in more efficient re-entry of the cells into the cell cycle after stress. Thus, the control of lncRNA expression as a new mechanism for the regulation of cell-cycle progression opens new avenues to understand how stress adaptation can be accomplished in response to changing environments.

## Cell-cycle progression upon osmostress

### Role of Cdc28 in cell-cycle control: CDK1 activity and association with cyclins

All cell-cycle events in budding yeast are biochemically coordinated, mostly by a single cyclin-dependent kinase (CDK), Cdc28 (Hartwell et al. 1973). Cdc28 activity and specificity are tightly controlled in a time-dependent manner through the regulation of different phase-specific cyclins, phosphorylations and inhibitors. The combination of multiple oscillatory mechanisms that collaborate in providing alternate periods of low and high levels of the different cyclin-CDK activities ensures orderly progression through the cell cycle (Spellman et al. 1998); (Orlando et al. 2008). In *S. cerevisiae*, Cdc28 cyclins have historically been classified into two broad groups: G1 cyclins (Cln1–3) that regulate events during the interval between mitosis and DNA replication and B-type cyclins (Clb1–6) that are required for replication, G2 progression and passage through Mitosis (Mendenhall and Hodge 1998). All cyclins except for Cln3 experience waves of production and destruction in pairs. Although cyclins show a high level of functional redundancy, the specificity of each cyclin is assured by a variety of mechanisms (Bloom and Cross 2007). Cyclins show different sensitivity to cell-cycle-regulated inhibitors, are restricted to different subcellular locations and are controlled in a time-dependent manner by specific transcriptional activators. Moreover, cyclins are degraded by phase-specific events, bind only to specific targets and facilitate different Cdc28 inhibitory phosphorylations when bound to it. Whereas the association of Cdc28 with cyclins and cyclin-dependent kinase inhibitor (CDKi) is a key regulatory element of Cdc28 kinase activity; the levels of Cdc28 protein do not change significantly during cell-cycle progression (Spellman et al. 1998).

#### Transcriptional control of the cell cycle

An astonishing amount of data has been generated over decades, which were aimed at understanding the complex regulatory network that governs cell-cycle progression. In the early nineties, mRNAs whose levels oscillated as yeast cells progressed thorough the cell cycle were identified (Price et al. 1991). Subsequently, the dynamics of cell cycle driven transcriptional waves were assessed using microarrays and these analyses significantly extended the number of known cell-cycle regulated genes to up to 800 genes (Cho et al. 1998; Spellman et al. 1998). Clustering of periodic transcripts led to the identification of cell-cycle regulated gene groups involved in cell-cycle control, budding, mating, DNA replication and repair. Cyclins are prototypical genes that display and determine induction of such periodic waves of transcription. Later studies devoted their efforts to understand the timing of these transcriptional oscillations by analyzing the genome-wide targets of the transcription factors that dictate cyclic specific expression (Lee et al. 2002; Simon et al. 2001). Consequently, a fully connected transcriptional circuit was delineated (Gauthier et al. 2008).

### Control of cell-cycle progression by the Hog1 SAPK

Stress-activated protein kinases (SAPKs) modulate cell-cycle progression and gene expression in response to extracellular insults. In yeast, osmostress induces activation of the p38-related SAPK Hog1 which leads to cell-cycle delay in different phases of the cell cycle (Saito and Posas 2012). Exposure to stress leads to a rapid but transient cell-cycle delay that depends on the strength of the stress and the degree of SAPK activation (Adrover et al. 2011). Long-term activation of Hog1 leads to a sustained cell-cycle arrest that, when maintained, provokes cells to enter into apoptosis (Vendrell et al. 2011). Hog1 controls cell-cycle progression in the G1, S and G2/M phases of the cell cycle, clearly indicating that, upon stress, cells need to transiently arrest cell-cycle progression for proper adaptation. Indeed, cells unable to arrest the cell cycle in the presence of stress become osmosensitive (Clotet et al. 2006; Duch et al. 2013; Escote et al. 2004).

#### Phosphorylation of core components of the cell-cycle machinery by Hog1

**Fig. 1:**
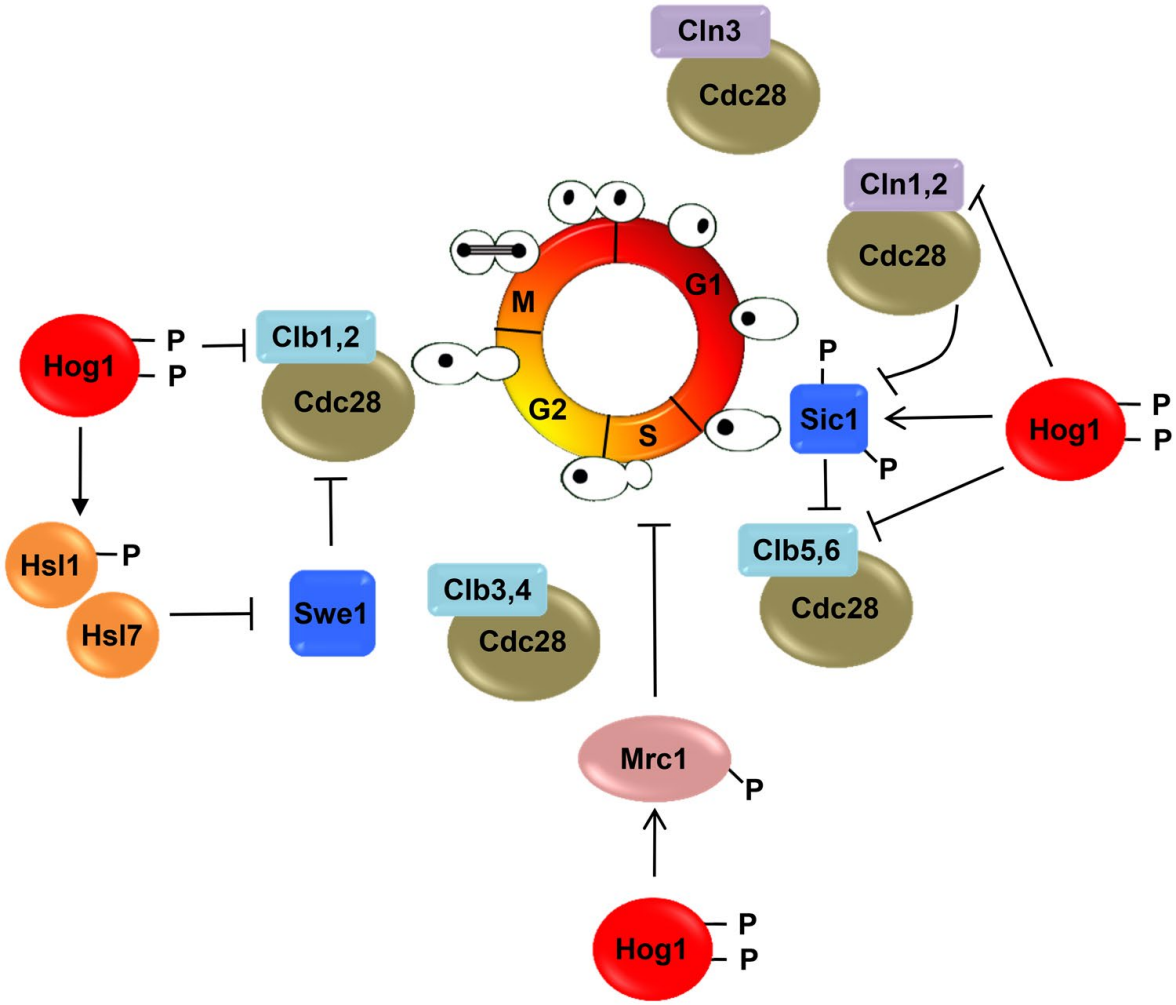
Control of cell-cycle progression by Hog1. The cyclin-dependent kinase (CDK) Cdc28 associates with phase-specific cyclins (shown around the *central circle*) to regulate passage through the cell cycle (G1 → S → G2 → M). Upon stress, Hog1 modulates progression at all phases of the cell cycle by acting on core elements of the cell cycle machinery

Activation of Hog1 leads to regulation of different key components of the cell-cycle machinery (Fig. 1). In G1, Hog1 directly phosphorylates the Sic1 CDKi promoting its stability. Hog1-phosphorylated Sic1 inhibits Cdc28–Clb5/6 complexes and prevents replication (Adrover et al. 2011; Escote et al. 2004). The lack of *SIC1* leads to genomic instability when cells are stressed in G1. During S phase, Hog1 phosphorylates Mrc1 a component of the replication complex that links the helicase with the DNA polymerase. Phosphorylation of Mrc1 leads to inhibition of origin firing as well as lowers down replication fork progression. Therefore, by delaying replication, Hog1 plays a key role in preventing conflicts between RNA and DNA polymerases. Hence, Mrc1 is a key protein for integration of signals from the DNA-damage checkpoint as well as of environmental signals mediated by Hog1 (Duch et al. 2012, 2013). In addition, during G2/M, Hog1 targets Hsl1 to delay cell-cycle progression via the inhibitory phosphorylation of Cdc28 by Swe1 (Clotet et al. 2006; Duch et al. 2012). The combined data suggest that, in the presence of stress, Hog1 impacts on basic components of the cell-cycle machinery to delay the cell cycle and to permit proper generation of adaptive responses before progression into the next phase of the cell cycle.

#### Regulation of CDK activity by the down-regulation of cyclin expression by Hog1

In addition to the direct phosphorylation of several components of the cell-cycle core machinery, Hog1 also regulates cell-cycle transitions by down-regulation of cyclin expression. In G1, activation of Hog1 prevents induction of *CLN1*, *CLN2* and *CLB5* cyclin genes (Adrover et al. 2011; Escote et al. 2004). Albeit the mechanism by which Hog1 represses expression from promoters regulated by G1 specific transcription factors, MBF and SBF, remains to be elucidated, mathematical modeling supported by quantitative in vivo experiments showed that *CLB5* down-regulation is a key regulator in G1 arrest whereas *CLN1*,*2* down-regulation or stabilization of Sic1 seems to be important only during late G1, when Hog1 control over Clb5 is not sufficiently tight to prevent S phase entry (Adrover et al. 2011). Therefore, the complex and strict Hog1 control over the G1/S network clearly illustrates the necessity of cell adaptation to osmostress prior to re-entering the cell cycle. Similarly, activation of Hog1 in G2 leads to down-regulation of the *CLB2* cyclin gene (Alexander et al. 2001; Clotet et al. 2006). The synergistic regulation by Hog1 of core elements of the cell cycle by direct phosphorylation, together with Hog1 down-regulation of cyclin expression, might serve to efficiently block cell-cycle progression in response to stress.

## Control of transcription in response to stress

**Fig. 2:**
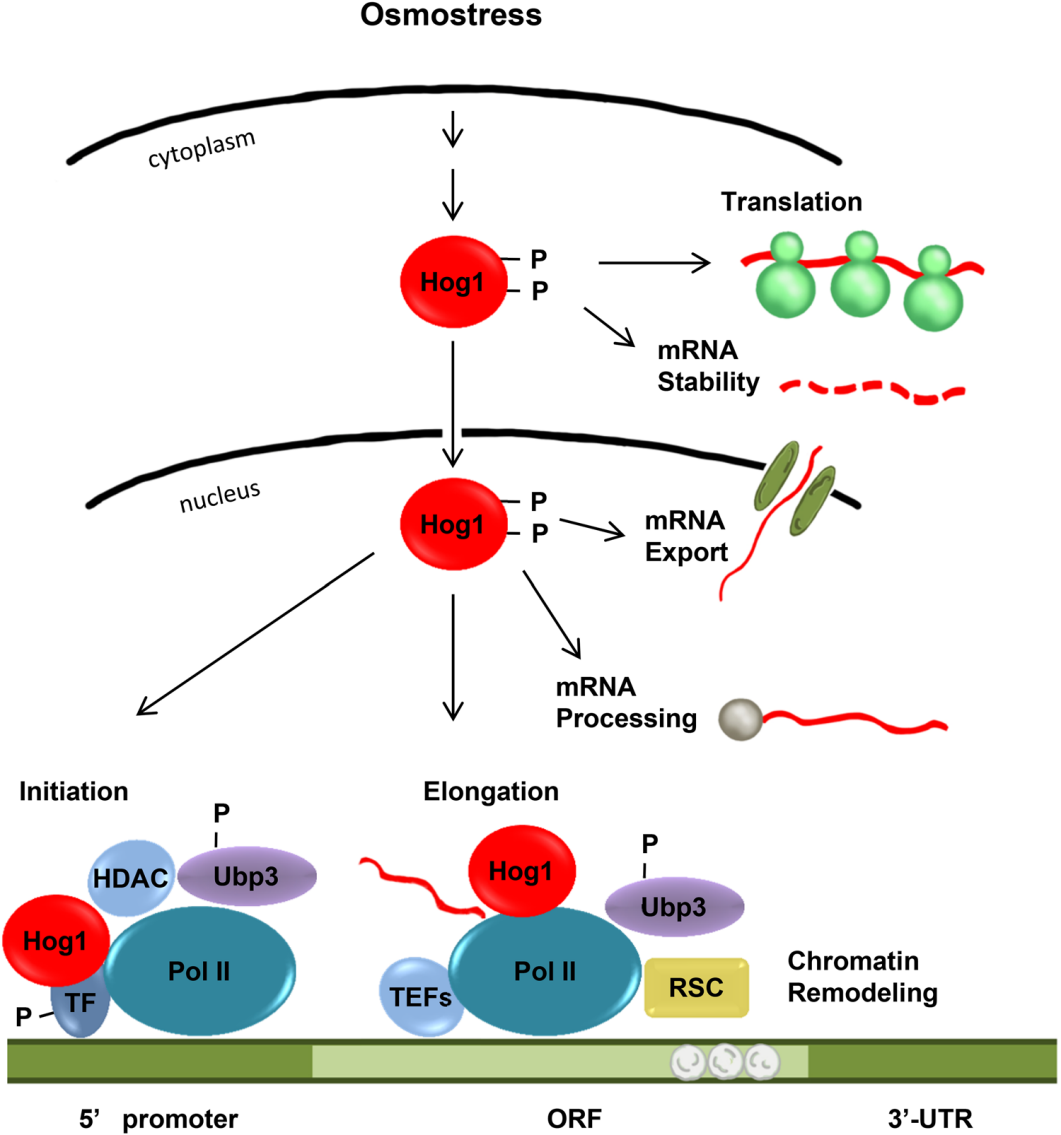
Control of mRNA biogenesis by the Hog1 MAPK. Once activated, Hog1 controls mRNA biogenesis in both the nucleus and the cytoplasm. In the nucleus, Hog1 associates with stress-responsive loci to modulate transcription initiation and elongation

Stress-activated protein kinases regulate gene expression to maximize cellular adaptation to environmental stresses (de Nadal et al. 2011; Weake and Workman 2010). Exposure to high osmolarity causes a sudden change in water activity and ionic strength, which impacts protein interactions. Most DNA binding proteins rapidly and transiently disassociate from chromatin upon osmostress (Proft and Struhl 2004). Moreover, a transcriptional burst of osmoresponsive genes occurs within minutes after stress. Transcriptional reprogramming is not essential for the short-term adaptive response but is crucial for long-term adaptation since mutants that display impaired transcription are unable to grow under osmostress (de Nadal et al. 2004; Mas et al. 2009; Zapater et al. 2007). Osmostress regulated genes have been implicated in carbohydrate metabolism, protein biosynthesis, general stress protection and signal transduction. Hog1 plays a key role in transcriptional reprogramming (Causton et al. 2001; Gasch et al. 2000; Pokholok et al. 2006; Posas et al. 2000; Rep et al. 2000) and coordinates the induction of osmoresponsive genes by controlling the entire process of mRNA biogenesis (de Nadal et al. 2011) (Fig. 2).

### Transcriptional reprogramming by Hog1 upon stress

The best characterized mechanism by which Hog1 controls transcription initiation is by direct phosphorylation of promoter-specific transcription factors (de Nadal et al. 2003; Proft and Struhl 2002; Ruiz-Roig et al. 2012). Additionally, Hog1 itself binds to chromatin through physical interaction with the transcription factors that serve as an anchoring platform for Hog1 (Alepuz et al. 2001; Pascual-Ahuir et al. 2006; Pokholok et al. 2006; Proft et al. 2006). Binding of Hog1 stimulates recruitment of the basic transcription machinery; Mediator, SAGA, SWI/SNF, Rpd3 deacetylase and Ubp3 deubiquitinase to promote transcription (Alepuz et al. 2003; de Nadal et al. 2004; Guha et al. 2007; Kobayashi et al. 2008; Proft and Struhl 2002; Sole et al. 2011; Zapater et al. 2007).

Hog1 is recruited not only to promoters but also to coding regions where it travels with elongating RNA Pol II and serves as a selective elongation factor for stress-responsive genes (Pokholok et al. 2006; Proft et al. 2006). Strong induction of gene expression in response to stress requires major changes in the chromatin structure of osmoresponsive genes (Nadal-Ribelles et al. 2012). To achieve efficient nucleosome eviction, Hog1 targets the RSC complex to code regions by directly binding to it (Mas et al. 2009). Repositioning of nucleosomes at stress-responsive genes is mediated by the INO80 complex (Klopf et al. 2009). Analysis of the signaling and transcription dynamics of the HOG pathway in single cells demonstrated that Hog1 activation increases linearly with osmostress, while transcriptional output exhibits a bimodal behavior. The cause for this stochastic expression is determined by the state and structure of chromatin (Neuert et al. 2013; Pelet et al. 2011; Zechner et al. 2012).

mRNA processing, stability and export of stress-responsive nascent transcripts are also regulated by Hog1. Following osmostress, the synthesis and half-life of osmoresponsive mRNAs increase while a broad range of other RNAs is concurrently destabilized (Miller et al. 2011; Molin et al. 2009; Romero-Santacreu et al. 2009). Moreover, in response to osmostress, Hog1 phosphorylates components of the inner nuclear basket (Nup1, Nup2 and Nup60), which associates with osmoresponsive promoters in a Hog1 activity-dependent manner (Regot et al. 2013).

### Genome-wide picture of the osmostress transcriptome

Although single case studies have provided very useful data, genome-wide approaches have provided a broader picture of the role of Hog1 as a master regulator of the massive transcription reprogramming that occurs in response to osmostress. DNA microarray studies have shown that 5–7 % of the protein coding genes show significant changes in their expression levels after mild osmotic shock. Depending on the severity of the stress, induction of up to 80 % of the induced transcripts depends on Hog1 (Capaldi et al. 2008; Causton et al. 2001; Gasch et al. 2000; O’Rourke and Herskowitz 2004; Posas et al. 2000; Rep et al. 2000). Other systematic analyses have been carried out to characterize the contribution of each stress-responsive transcription factor controlled by Hog1 to the total transcriptional response. Transcription profiling of individual or multiple mutants of transcription factors used by Hog1 shed light on the complexity of the transcription factor network (Capaldi et al. 2008; Ni et al. 2009). Further studies using high density oligonucleotide or tiling arrays (see below) complemented and deepened our knowledge of the genome-wide picture of the osmostress transcriptome.

Transcription rate in response to osmostress has been measured using two different techniques: genomic run-on (GRO) and dynamic transcription analysis (DTA) (Miller et al. 2011; Romero-Santacreu et al. 2009). Both types of studies identified changes in mRNA synthesis and decay in response to osmostress and identified three phases of the stress response: shock, induction and recovery phases (Miller et al. 2011). During the initial shock, synthesis and decay rates globally decrease causing storage of mRNA in P-bodies (Romero-Santacreu et al. 2009). Later, in the induction stage, the mRNA synthesis rates of osmoresponsive genes increase together with mRNA decay rates to ensure high production and removal of stress mRNAs. In the subsequent recovery phase, mRNA decay and synthesis are restored to prestimulation levels (Miller et al. 2011).

Genome-wide localization of Hog1 and stress-responsive transcription factors was assessed by combining chromatin immunoprecipitation with microarray technology (ChIP on chip) (Capaldi et al. 2008; Proft et al. 2006). Moreover, an attempt was made to determine the role of Hog1 in the genome-wide distribution of RNA Pol II using ChIP followed by deep sequencing (ChIP-seq) analysis (Cook and O’Shea 2012; Nadal-Ribelles et al. 2012). In response to osmostress, there is a genome-wide tendency to lose association of RNA Pol II with the genome, which leads the entire genome into a repressive state. On the other hand, while the entire genome is undergoing RNA Pol II dissociation, Hog1 binds through specific transcription factors to selectively target RNA Pol II to induce stress-responsive genes. Thus, colocalization of Hog1 and RNA Pol II bypasses the osmostress-induced global transcription repression. These data are consistent with single case studies. Osmoresponsive transcription is characterized by strong induction of gene expression from almost no expression under basal conditions to maximal activation (fold changes ranging from 5 to 200) in just 10 min (Posas et al. 2000). This process involves major changes in chromatin structure, which, for osmoresponsive genes, require the presence of Hog1 (Mas et al. 2009). Genome-wide changes in chromatin structure in response to osmostress were determined using micrococcal nuclease followed by deep sequencing (MNase-seq). Hog1-dependent genes displayed massive nucleosome eviction at promoter and coding regions in response to stress that was completely dependent on the presence of Hog1 (Nadal-Ribelles et al. 2012).

The global snapshot of gene expression, protein localization and nucleosome occupancy that was thus obtained revealed a dose-dependent correlation between chromatin remodeling and both the strength and the residence time of Hog1 at target genes, making transcription of these genes more efficient. In other words, Hog1 bypasses stress-mediated down-regulation of transcription via its effects on RNA polymerase II redistribution and chromatin remodeling.

## Induction of a new set of lncRNAs in response to environmental insults

### Pervasive transcription and lncRNAs

Expression profiling using microarrays has been highly efficient in obtaining quantitative measurements of total mRNA (Young 2000). However, this method cannot fully unravel the complexity of the transcriptome. To overcome these limitations, several new methods with increased sensitivity and coverage have been developed (i.e. tiling arrays, RNA-seq, GRO-seq, NET-seq and TIF-seq). These unbiased strategies interrogate both strands of the genome allowing the detection of new transcripts such as non-coding RNAs (ncRNAs) and complex transcriptional architectures (Bertone et al. 2004; Nagalakshmi et al. 2008; Garcia-Martinez et al. 2004; Churchman and Weissman 2011; Pelechano et al. 2013). Of this newly described plethora of RNA species, the best characterized in structure and function are the short ncRNAs (<200 nt in length) that encompass several classes such as: small interfering RNAs (siRNAs), micro RNAs (micRNAs) and PIWI-interacting RNAs (piRNAs). The role of short ncRNAs as powerful regulators of gene expression is well accepted and established in complex organisms reviewed in Moazed (2009). On the other hand, long noncoding RNAs (lncRNAs, >200 nt in length) have also been identified in virtually all studied organisms regardless of genome size and complexity. However, our understanding of their properties is frequently descriptive (i.e. size, stability and genomic location), and in most cases their function has not been assessed (Pelechano and Steinmetz 2013).

In *S. cerevisiae* as much as 85 % of the genome is expressed under basal conditions, including a large number of lncRNA transcripts (David et al. 2006). Further studies have identified and characterized new lncRNA families based on their stability. Thus, cryptic unstable transcripts (CUTs) are noncoding RNAs that are only expressed in mutants of the nuclear exosome (*rrp6*), Xrn1-sensitive unstable transcripts (XUTs) are noncoding transcripts that are expressed in cytosolic exosome mutants (*xrn1*), and stable unannotated transcripts (SUTs) are a group of stable non-coding RNAs (van Dijk et al. 2011; Xu et al. 2009). Recently, other groups reported the appearance of novel ncRNA transcripts in the deacetylase Set3 deletion strain (Kim et al. 2012) and in the absence of gene looping (in a *ssu72* mutant) that have been named Ssu72-restricted transcripts (SRTs) (Tan-Wong et al. 2012).

### Change of the noncoding transcriptome upon environmental changes

The transcription of lncRNAs was initially interpreted as transcriptional noise or as a by-product. However, several studies in yeast have started to shed some light on the complexity of the stress-responsive transcriptome under several conditions such as growth in different fermentable carbon sources (Xu et al. 2009), diauxic shift (Kim et al. 2012) and in response to oxidative stress in fission yeast (Quintales et al. 2010). Therefore, adaptive stress responses also require regulation of the noncoding transcriptome. Upon osmostress, Hog1 induces the expression of a complete new set of lncRNAs (Nadal-Ribelles et al. 2014).

## Cdc28 is regulated by a stress-induced lncRNA

### A new set of lncRNAs is induced upon stress

The use of tiling arrays has shown that in addition to coding genes, around 200 lncRNAs are strongly and rapidly induced upon osmostress, of which 91 are Hog1 dependent. The promoters of these stress-responsive lncRNAs show similarities with those of stress-responsive genes. Hog1 associates with these promoters and stimulates the recruitment of RNA Pol II (Nadal-Ribelles et al. 2014). Functional characterization of these stress-induced lncRNAs showed that there is a significant number of genes that display a positive correlation between expression of the sense and antisense-induced (from 8 to 41, depending on the analysis method). One of these genes that shows a clear correlation between the sense mRNA and antisense lncRNA expression is *CDC28,* which encodes the main CDK that drives progression of the cell cycle in yeast.

### An lncRNA up regulates Cdc28 protein levels to promote cell-cycle re-entry in the presence of osmostress

**Fig. 3:**
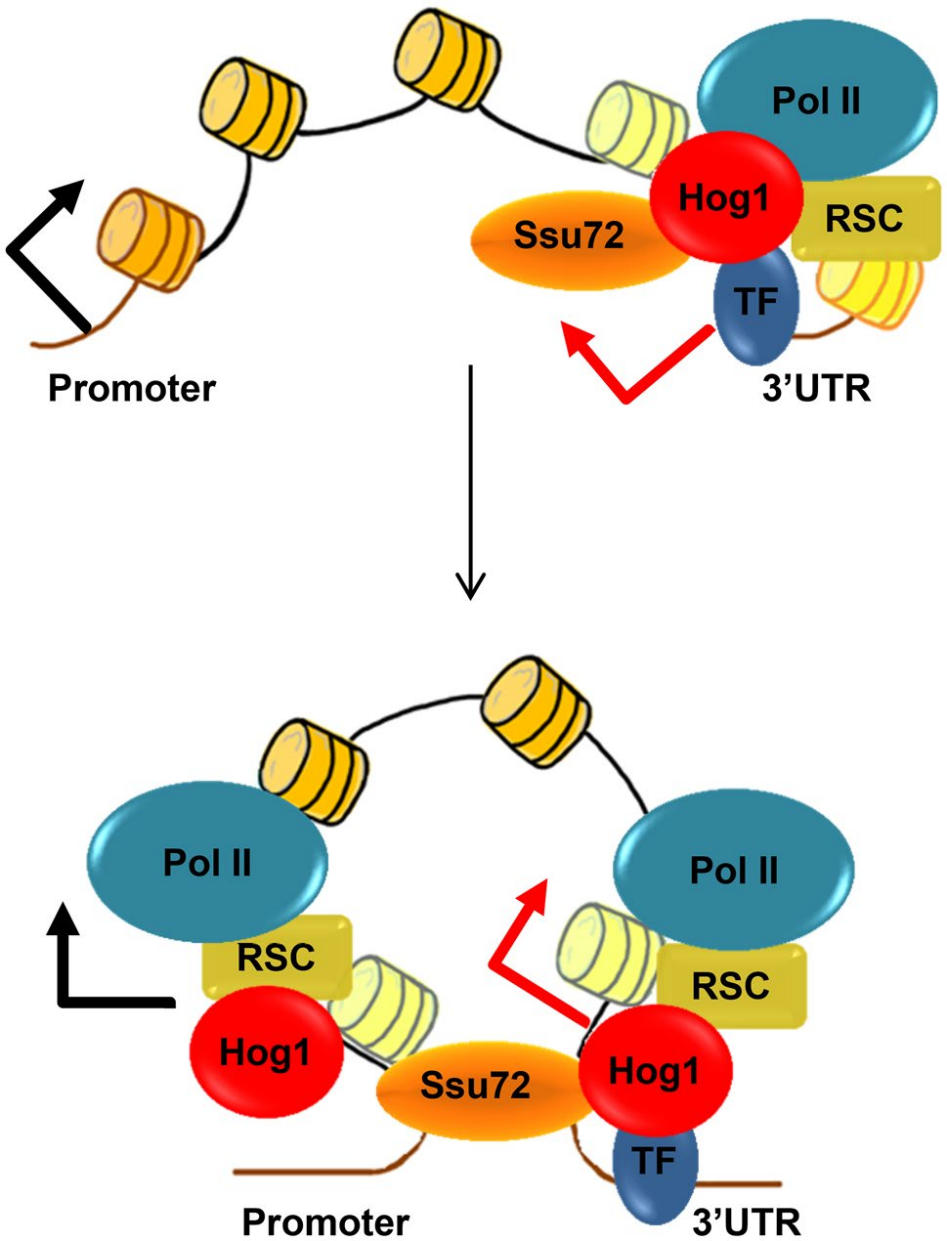
Schematic representation of the two-step model of lncRNA and gene looping mediated gene regulation. Hog1-mediated regulation of sense (*black arrow*) and antisense lncRNA (*red arrow*) transcripts following osmostress. *Top panel* Stress induces Hog1-mediated recruitment of transcriptional machinery to the 3′UTR region of an osmostress regulated gene. *Bottom panel* The resulting induced lncRNA, in the presence of Ssu72, causes looping of the DNA and translocation of Hog1 and RSC to the +1 nucleosome region, resulting in enhanced sense transcription in addition to antisense transcripts. *Light colored* nucleosomes indicate remodeled nucleosomes

Genetic and biochemical characterization of the induction of *CDC28* gene expression by Hog1-induced antisense lncRNA led to the following tentative mechanistic model. Upon osmostress, Hog1 associates with the 3′ UTR region of *CDC28,* via its association with Sko1 (unpublished data), and induces lncRNA transcription. This induction of lncRNA permits the establishment of gene looping mediated by Ssu72. Gene looping leads to the transfer of Hog1 to the +1 nucleosome region of *CDC28*. Association of Hog1 with the proximal region of the gene serves to target the RSC chromatin remodeler, which remodels the +1 region, thus permitting an increase in transcription of the *CDC28* gene (Fig. 3).

The increase in *CDC28* expression upon stress results in an increase in *de novo* synthesis of Cdc28. As described above (Sect. Control of cell cycle progression by the Hog1 SAPK), Hog1 mediates a rapid, but transient, arrest of cell-cycle progression to allow adaptation. Mechanisms under the control of Hog1 include the down-regulation of cyclins and the direct inhibitory phosphorylation of Swe1 to reduce Cdc28 activity. These down-regulatory effects of Hog1 on Cdc28 appear to be in contradiction to an increase in Cdc28 protein. However, the actual increase in Cdc28 levels does not occur until about 30 min after the cells are subjected to 0.4 M NaCl. This increase in Cdc28 protein coincides with the restart of the cell cycle after stress-induced arrest, which suggests a role for the increase in Cdc28 levels during the recovery phase. Of note, although cells deficient in *CDC28* lncRNA underwent arrest similar to wild-type cells upon stress, they were less efficient in re-entering the cell cycle, suggesting that the increase in Cdc28 protein permits a faster recovery from the cell cycle delay that is caused by stress. Therefore, Hog1 is able both to induce a cell-cycle delay and to promote cell recovery by controlling Cdc28 by different mechanisms with a different temporal outcome.

## Control of the cell cycle by lncRNAs

### Cell-cycle oscillation of coding and lncRNAs

The establishment of a critical role for a lncRNA in *CDC28* and cell-cycle control has prompted us to analyze other lncRNAs. Deep transcriptome analyses have highlighted the complexity of gene expression networks. Even in scenarios such as the cell cycle that had been previously studied in depth, tiling array analyses have extended the number of periodically expressed protein coding genes and have provided evidence of an additional layer of regulation based on lncRNAs (Granovskaia et al. 2010).

**Fig. 4:**
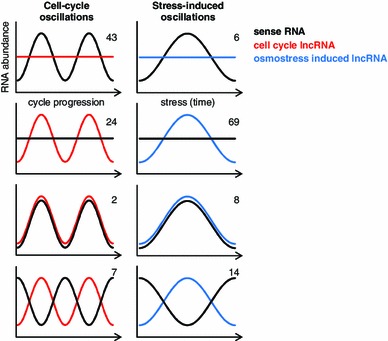
Types of dynamics of sense-antisense RNA pairs. Schematic representation of expression profiles of sense-antisense RNAs during cell-cycle progression (*left panels*) and under osmostress conditions (*right panels*). *Numbers* in the *upper corner* of each graph indicate the number of cases described in the literature for the mitotic cell cycle (Granovskaia et al. 2010) and for stress conditions (Nadal-Ribelles et al. 2014, unpublished data)

Depending on whether the sense or the antisense RNA is induced, there are four possible scenarios that can describe their transcriptional relationship (Fig. 4). The first scenario, which is the most common one for cell-cycle regulated pairs, is where sense RNA is induced but lncRNA remains constant. In the second scenario, sense RNA remains constant but lncRNA expression is induced. This scenario is the most common one for osmoresponsive lncRNAs. The third category is exemplified by *CDC28* that displays the most interesting pattern in which both sense and antisense RNA expression increase with the same kinetics and times. The final scenario is where sense and antisense RNA expression increase in an oscillatory anti-correlation pattern.

Regulation of some key cell-cycle regulatory proteins falls into this last behavioral category and probably the most striking case is that of the CDK inhibitor *FAR1*. Expression of *FAR1* lncRNA peaks during the G1/S transition, when Far1 levels should be very low to ensure entry into replication. Of note, in response to osmostress the HOG pathway controls the expression of *FAR1* lncRNA that anti-correlates with the sense transcript (Nadal-Ribelles et al. 2014). The mechanism of action of this sense-antisense RNA pair remains unknown although the potential of regulating CDK inhibitors by a number of non-post-translational mechanisms might offer a higher degree of flexibility or contribute to the robustness of the response. Interestingly, in response to stress and cell-cycle progression, there is an additional class of regulated intergenic non-coding RNAs, although the physiological role, if any, of these RNAs remain obscure (Granovskaia et al. 2010; Nadal-Ribelles et al. 2014).

The presence of lncRNAs in tightly coordinated and complex networks such as the mitotic cell cycle and the response to osmostress suggests a regulatory action of antisense transcripts. Examples such as the ones described above illustrate the potential extent of lncRNA function.

### Different examples of lncRNAs that target cell-cycle genes (cyclins, CDKs and CDK inhibitors)

Phenotypic variation cannot be satisfactorily explained by protein coding genes alone. Interestingly, analysis of fully sequenced genomes has shown that the number of non-protein coding sequences consistently increases with organism complexity (Taft et al. 2007). Robustness of the cell-cycle network is essential for the survival of all organisms; indeed, lncRNAs that affect the cell-cycle have been identified in a variety of organisms from prokaryotes to higher eukaryotes.

For example, replication initiation in bacteria is conducted by the replication initiation factor (DnaA) that recognizes replication origin (*ori*) and triggers the assembly of the replisome. The mechanism of replication (DnaA and its binding sites) is conserved throughout the prokaryotic kingdom. Recently, the existence of an RNA that was encoded in cis to the *DnaA* gene was shown to regulate the stability of the sense mRNA in response to environmental insults including osmostress in *Salmonella typhi*. Expression of the antisense RNA increases upon osmotic shock and causes a concomitant increase in *DnaA* mRNA stability, thereby enhancing total protein DnaA levels that ultimately help cells to recover from such stresses (Dadzie et al. 2013). This scenario resembles the *CDC28* case in yeast.

In higher eukaryotes lncRNAs also target master regulators of the cell cycle by acting through several mechanisms that operate as regulators of a variety of features such as epigenetic state, transcription factors and post-transcriptional events reviewed in Kitagawa et al. (2013). An interesting example is the antisense RNA ANRIL that is induced by the DNA-damage signaling pathway (ATM-E2F1) (Wan et al. 2013). Expression of ANRIL causes an epigenetic repression of the INK locus where the p15 and p16 CDK inhibitors are located and ANRIL is critical for cell-cycle regulation and tumor progression (Kotake et al. 2011; Yap et al. 2010).

## Perspective

The presence of lncRNA transcription provides an exciting landscape of new possibilities in the field of transcription in eukaryotes and challenges the classical view of gene unit. The mechanisms of lncRNA regulation of the transcription of protein coding genes are still far from being understood, mainly because detailed biochemical studies are scarce. The furthering of our knowledge in the direction of understanding of these mechanisms will allow assessment of whether specialized lncRNA transcription machinery exists (or of the requirements for lncRNA transcription). Knowledge of the transcriptional regulation, the structure and the stability of lncRNAs may indicate a new level of transcriptional or post-transcriptional control for the modulation of cell physiology. The examples described above illustrate the potential extent of the regulatory actions of lncRNA transcripts, which range from their role in cellular adaptation to stress to their role in the control of cell-cycle progression. Analysis at the single cell level will lead to an understanding of the complex dynamic interplay between sense and antisense RNA that may be masked by population-based approaches. Determination of whether sense-antisense RNA pairs coexist or are mutually exclusive will help to dissect the impact of lncRNA transcription on the transcription of protein coding genes. Major physiological roles have already been assigned to lncRNAs (Kitagawa et al. 2013; Ng et al. 2013; Bergmann and Spector 2014; Rossi and Antonangeli 2014) and the enormous variety of mechanisms by which they modulate those phenotypes are only beginning to be understood (Pelechano and Steinmetz 2013; Vance and Ponting 2014).
